# The miRNA Argonaute protein, ALG-2, maintains neurobehaviors in adult *C. elegans*

**DOI:** 10.17912/micropub.biology.002217

**Published:** 2026-08-09

**Authors:** Ava Chon, Runtian Jiang, Yuxuan Guo, Yamin K. Phyu, Lilly M. Goldberg, Erin C. Schiksnis, Amy E. Pasquinelli, Sierra D. Palumbos

**Affiliations:** 1 Biology, Swarthmore College, Swarthmore, PA, United States; 2 Molecular Biology, UC San Diego, San Diego, CA, United States

## Abstract

microRNAs (miRNAs) are short, non-coding RNAs essential for gene regulation in many different processes, including neuronal development. However, the role of the miRNA pathway in maintaining neuronal health throughout aging is less understood. Here, we ask how the miRNA pathway in adulthood impacts neurobehaviors in *C. elegans* . Argonaute-like Gene 2 ( ALG-2 ) is a protein required for the accumulation and function of certain miRNAs in *C. elegans* . Using the auxin-inducible degron 2 (AID2) system for temporal knockdown, we demonstrate that the miRNA Argonaute protein, ALG-2 , is required throughout adulthood to maintain two well-characterized neurobehaviors, basal slowing response and mechanosensation.

**Figure 1. ALG-2 function in adulthood required for neurobehaviors:** **A)** Graphic depicting insertion of AID2 at the *alg-2* gene locus. **B)** Immunoblot of ALG-2 protein levels from PQ668 worms following 30 minutes of auxin treatment at ranging auxin concentrations. Actin was probed as positive control. **C)** BSR assay of day 1 adult PQ668 strain and **D)** day 4 adult PQ668 and N2 strains. For each condition, n=27. BSR was calculated by subtracting the number of body bends in the presence of food from the number of body bends when food was absent. One-way ANOVA with multiple comparisons used to determine significance; *** indicates p<0.001. **E)** Soft touch response assay of day 2 adult PQ668 and N2 strains with or without auxin treatment. N=30 worms for each condition across 3 trials. One-way ANOVA test with multiple comparisons was used to determine significance, *** indicates p<0.001. **F)** Chemotaxis Index (CI) of day 2 adult PQ668 and N2 *C.elegans* with and without auxin treatment in response to isoamyl alcohol (IA). CI was calculated as the number of worms at IA minus the number of worms at H₂O, divided by the total number of worms. Error bars represent SD. n ≥10 per trial, 3 trials. One-way ANOVA with multiple comparisons used to determine significance. **G)** *alg-2* expression in transcripts per million (TPM) at L1, L4 and Adult stages in touch receptor neurons. Data taken from single cell data reported by the *C. elegans* Neuronal Gene Expression Map & Network (Taylor et al., 2021).

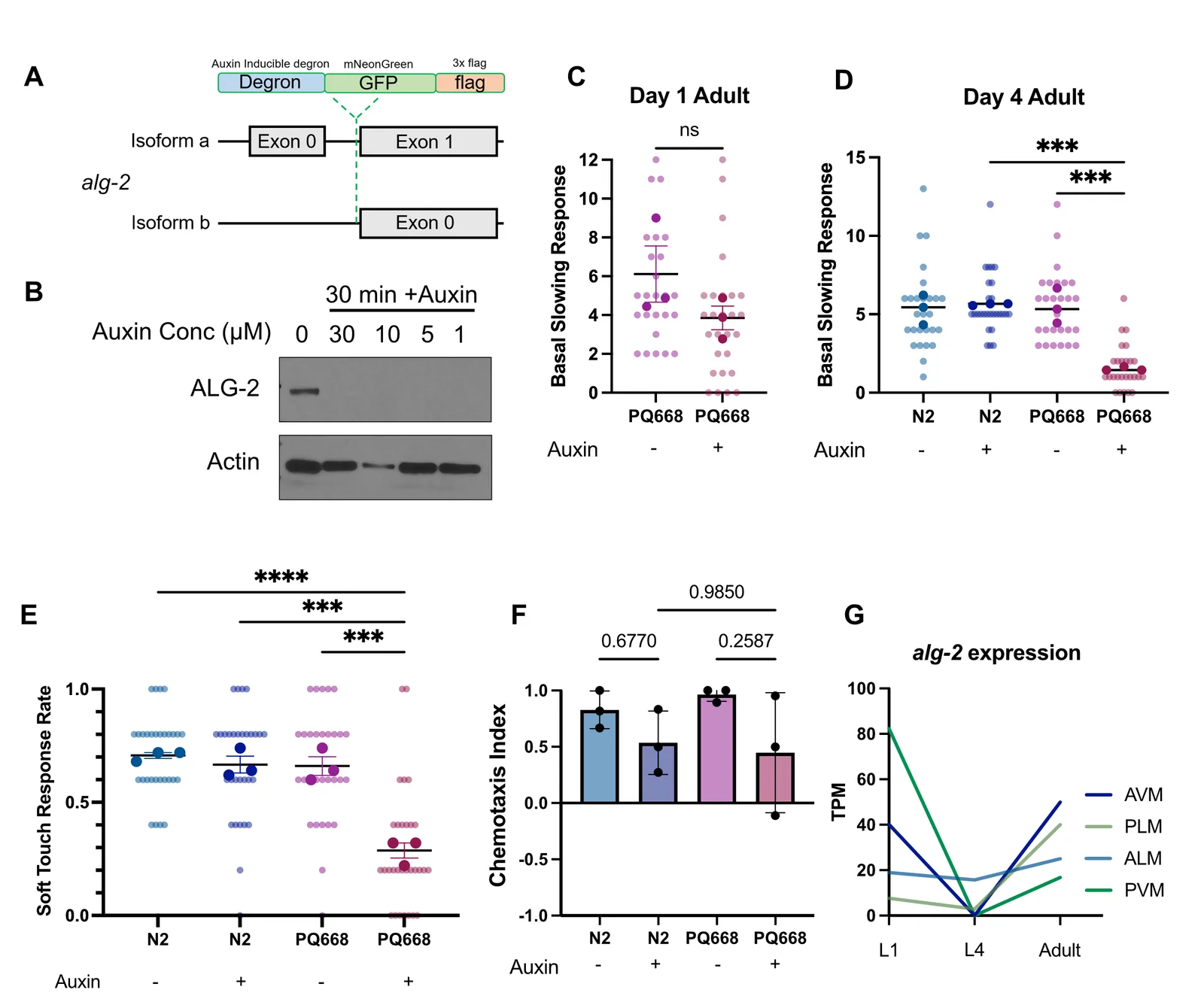



## Description

miRNAs are non-coding RNAs that are approximately 22 nucleotides long which repress gene expression of target mRNAs (Shang et al., 2023). miRNAs associate with an Argonaute protein, forming the miRNA-induced silencing complex (miRISC), which recognizes specific mRNA targets and induces their translational repression and degradation (Shang et al., 2023, Sala et al., 2020). *C. elegans* express two argonaute proteins that mediate miRNA repression, ALG-1 and ALG-2 , which exhibit distinct roles (Tops et al., 2006, Aalto et al., 2018). While miRNAs are well characterized in development (Ivey and Srivastava, 2015), their role in aging and neurodegeneration is still an emerging field (Elder and Pasquinelli, 2022). Here, we ask whether the miRNA pathway has a role in maintaining neuronal homeostasis in adult *C. elegans* by tracking common neurobehaviors (Caldwell et al., 2020).

To do this, we used CRISPR/Cas9 to insert an Auxin-inducible degron 2 sequence at the N-terminus of *alg-2* coding sequence, to generate a worm strain, PQ668 (AID2::mNeonGreen::3Xflag:: alg-2 ) where we could temporally regulate *alg-2* expression in adult *C. elegans* via the exogenous addition of auxin ( Fig. 1A ) (Nishimura et al., 2009, Dickinson et al., 2015, Yesbolatova et al., 2020). To confirm successful knockdown of ALG-2 , we tracked ALG-2 protein levels using immunoblotting from worms treated with and without auxin ( Fig. 1B ). Following as short as 30 minutes on auxin-plates, ALG-2 protein levels were reduced below detection. This was also confirmed by tracking the inserted GFP fluorescent reporter. Thus, we developed a tool where ALG-2 levels could be selectively reduced during adulthood, allowing us to ask if loss of ALG-2 impacted neuronal homeostasis.

*C. elegans* reduce their speed in the presence of food, a phenomenon termed Basal Slowing Response (BSR) (Rivard et al., 2010), which is dependent on dopaminergic transmission (Sawin et al., 2000). We measured BSR in control (PQ668 – auxin) and ALG-2 depleted worms (PQ668 + auxin) to ask how impairment of the ALG-2-miRNA pathway impacts dopaminergic transmission in *C. elegans .* In day 1 adult worms, there was no statistically significant difference in BSR between PQ668 - auxin and PQ668 + auxin worms (p=0.2244) ( Fig. 1C ). However, following 4 days without ALG-2 , PQ668 + auxin worms showed significantly reduced BSR compared to their matched controls (1.5185 vs. 5.4815, p=0.0008) ( Fig. 1D ). To confirm this observed effect was due to ALG-2 depletion and not the presence of auxin, we measured BSR in N2 worms in the presence or absence of auxin and observed no difference (p=0.9559) ( Fig. 1E ). Thus, we observed an age-dependent defect in BSR, suggesting a role for ALG-2 in maintaining dopaminergic transmission in adulthood.

We next asked whether other behaviors were impacted. *C. elegans* depend on soft touch sensation to navigate their world. Soft touch is communicated via mechanoreceptors expressed by six glutamatergic touch receptor neurons located in either the anterior or posterior of the worm (Chen and Chalfie, 2014). To determine whether depletion of ALG-2 in adulthood affects mechanosensation, we monitored soft touch response in Day 2 adult worms with control levels of ALG-1 (PQ668 - auxin) and those with ALG-2 depletion (PQ668 + auxin, L4-Day 2) following alternating anterior and posterior stimulation with an eyelash pick. While we observed a significant reduction in overall responses (64.33% vs. 83.00%, p=0.002), we noted that failure to respond was only observed following anterior stimuli. We therefore quantified anterior soft touch response and noted ALG-2 depleted worms (PQ668 + auxin) exhibited a defect in anterior mechanosensation compared to untreated controls (PQ668 - auxin) (28.67% vs. 66.00%, p=0.0002) ( Fig. 1E ). To determine whether auxin itself affected mechanosensation, we repeated the assay using N2 worms and found no significant difference between N2 worms grown on auxin vs. control plates (p=0.8283) ( Fig. 1E ). Our findings suggest that the miRNA Argonaute protein, ALG-2 , is selectively required for anterior mechanosensation in adult animals, potentially through maintaining glutamatergic transmission in a subset of touch receptor neurons.

*C. elegans* possess a well-organized chemosensory system that allows them to navigate a variety of olfactory and gustatory cues associated with food, danger, and mates (Bargmann, 2006). We used the well characterized attractant, isoamyl alcohol, (Bargmann et al., 1993) to ask if ALG-2 function in adulthood is similarly required for chemotaxis. We tracked the chemotaxis index in PQ668 and N2 wild type worms (day 2 adults) with and without auxin. In contrast to mechanosensation and BSR, we found no significant change in chemosensory behavior across any conditions ( Fig. 1F ). It is of note that both our auxin groups had higher variability in their response, suggesting that auxin itself could be impairing chemotaxis. Thus, loss of ALG-2 function did not impair chemotaxis, suggesting that distinct circuits are impacted by loss of ALG-2 activity.

Given our observations that adult expression of ALG-2 is selectively required for specific neurobehaviors (BSR and anterior mechanosensation), but not others (chemosensation) we analyzed *alg-2* expression dynamics in touch receptor and dopaminergic neurons across developmental stages using the *C. elegans* Neuronal Gene Expression Map & Network (Taylor et al., 2021). Touch receptor neurons (ALM, AVM, PLM, PVM) displayed a biphasic developmental regulatory pattern of *alg-2* expression (high in L1, low in L4, high in adults) ( Figure 1G ). As this developmental expression pattern was true in both anterior and posterior touch neurons, local *alg-2* transcription alone does not explain selective defect in anterior soft touch response. Similarly, *alg-2* expression in dopaminergic neurons was inconsistent with a simple cell-autonomous mechanism. While adult CEP neurons showed a modest increase in *alg-2* , ADE and PDE expressed *alg-2* at levels far below their developmental peaks. Given that miRNAs can be secreted and act extracellularly to impact protein expression in neighboring cells (Shang et al., 2023, Alkhazaali-Ali et al., 2024, Palumbos et al., 2025), we hypothesize that ALG-2 regulates neurotransmission, at least in part, in a non-cell-autonomous manner during aging. In summary, these results demonstrate that ALG-2 is required during adulthood to maintain glutamatergic and dopaminergic neurotransmission, suggesting miRNAs play an ongoing role in preserving neural function during aging.

## Methods

**Strain Preparation:** The N2 strain was obtained from the Caenorhabditis Genetics Center. The PQ668 (AID2::mNeonGreen::3Xflag:: alg-2 ) strain was developed using CRISPR-Cas9 to insert AID2::mNeonGreen::3Xflag before the 5' end of *alg-2* before exon 1 of isoform A and exon 0 of isoform B (See Jiang, 2022). Briefly, four plasmids were injected into young adult N2 worms: 1) 50ng/ul of homologous repair template (AID2::mNeonGreen::3xflag with ALG-2 homology arms), 2) 50ng/ul of pJB53 (Cas9 plasmid with ALG-2 specific sgRNA, modified from pJW1219, Addgene #61250), 3) 10ng/ul pGH8 (Prab-3::mCherry:: unc-54 3'-UTR, Addgene plasmid #19359) and 4) 5ng/μL pCFJ104 (Pmyo-3::mCherry:: unc-54 3'-UTR, Addgene plasmid #19328). Recombinant worms were isolated as previously described (Dickinson et al. 2015) and then backcrossed 3X to N2 to generate PQ668.

**Auxin Plates:** Nematode growth media (NGM) plates were prepared as described previously (Stiernagle, 2006). Auxin-containing NGM plates were prepared by adding 0.89 mg/mL auxin after autoclaving (Sharma et al., 2024). Plates were seeded with OP50 for 48 hours.

**Western Blot:** Western blot was carried out as described previously and ALG-2 was detected based on 3X flag insertion using anti-FLAG antibody (Van Wynsberghe et al., 2011).

**Soft Touch Response:** L4 PQ668 *C. elegans* were transferred to NGM plates with or without auxin and maintained at 23°C for 2 days. Adult Day 2 worms were transferred to an unseeded NGM plate and allowed to acclimate before testing. Gentle mechanical stimuli were applied using an eyelash pick by alternately stroking the anterior of the worm (posterior to the pharynx) and the posterior of the worm (anterior of the anus) for a total of 10 touches per worm (Chalfie et al., 2018). Responses to anterior touch were scored as reversals, whereas responses to posterior touch were scored as forward movement. Failure to produce a movement response following soft touch stimulation was scored as defective.

**Basal Slowing Response:** L4 *C. elegans* were plated on NGM plates with or without auxin. Plates were kept at 23℃ and worms were allowed to grow for either 1 or 4 days before being transferred to an unseeded NGM plate for analysis. After a 3-minute acclimation period, body bends were counted over a 20-second period. Worms were transferred to a seeded plate and the same procedure was repeated (Petratou et al., 2024). BSR was calculated for individual worms by subtracting the number of body bends in the presence of food from the number of body bends when no food was present.

**Chemotaxis Assay:** L4 *C. elegans* were transferred to OP50-seeded NGM plates with or without auxin and maintained at 23℃ for 2 days. Chemotaxis assay plates were prepared by dividing each plate into two halves and 5μL of isoamyl alcohol (IA) on one side and 5μL of deionized water (H₂O) on the other. 5μL of 0.5 M sodium azide was spotted on IA and H₂O to paralyze the worms upon contact. Day-2 adult worms were washed with 1 mL of M9 buffer (3 g KH₂PO₄, 6 g Na₂HPO₄, 5 g NaCl, 1 mL 1M MgSO₄) before being transferred to chemotaxis plates for 1-hour. A minimum of 10 worms per plate was assayed across 3 trials. Worms were manually counted under a dissecting microscope. The chemotaxis index was calculated as CI = (# of worms at IA − # of worms at H₂O) / total # of worms, and ranged from +1 (maximum attraction) to -1 (maximum repulsion) (Bargmann et al., 1993).

**Statistics** :

All statistical analyses were performed using GraphPad Prism (ver. 10) and specific tests are specified in figure legend.

## References

[R1] Aalto Antti P., Nicastro Ian A., Broughton James P., Chipman Laura B., Schreiner William P., Chen Jerry S., Pasquinelli Amy E. (2018). Opposing roles of microRNA Argonautes during Caenorhabditis elegans aging. PLOS Genetics.

[R2] Alkhazaali-Ali Zahraa, Sahab-Negah Sajad, Boroumand Amir Reza, Tavakol-Afshari Jalil (2024). MicroRNA (miRNA) as a biomarker for diagnosis, prognosis, and therapeutics molecules in neurodegenerative disease. Biomedicine & Pharmacotherapy.

[R3] Bargmann Cornelia I., Hartwieg Erika, Horvitz H. Robert (1993). Odorant-selective genes and neurons mediate olfaction in C. elegans. Cell.

[R4] Bargmann CI (2006). Chemosensation in C. elegans.. WormBook.

[R5] Caldwell Kim A., Willicott Corey W., Caldwell Guy A. (2020). Modeling neurodegeneration in
*Caenorhabditis*
 
*elegans*. Disease Models & Mechanisms.

[R6] Chalfie M, Hart AC, Rankin CH, Goodman MB (2014). Assaying mechanosensation.. WormBook.

[R7] Chen Xiaoyin, Chalfie Martin (2014). Modulation of
*C. elegans*
Touch Sensitivity Is Integrated at Multiple Levels. The Journal of Neuroscience.

[R8] Cinar Hulusi, Keles Sunduz, Jin Yishi (2005). Expression Profiling of GABAergic Motor Neurons in Caenorhabditis elegans. Current Biology.

[R9] Dickinson Daniel J, Pani Ariel M, Heppert Jennifer K, Higgins Christopher D, Goldstein Bob (2015). Streamlined Genome Engineering with a Self-Excising Drug Selection Cassette. Genetics.

[R10] Elder Corrina R., Pasquinelli Amy E. (2022). New Roles for MicroRNAs in Old Worms. Frontiers in Aging.

[R11] Ivey Kathryn N., Srivastava Deepak (2015). microRNAs as Developmental Regulators. Cold Spring Harbor Perspectives in Biology.

[R12] Jiang, R. 2022. Conditional depletion of ALG-1 and ALG-2 using auxin inducible degron 2 (AID2). *UC San Diego* . ProQuest ID: Jiang_ucsd_0033M_21343. Merritt ID: ark:/13030/m5hx8j4s. Retrieved from https://escholarship.org/uc/item/8646322z

[R13] Li Wei, Kang Lijun, Piggott Beverly J., Feng Zhaoyang, Xu X.Z. Shawn (2011). The neural circuits and sensory channels mediating harsh touch sensation in Caenorhabditis elegans. Nature Communications.

[R14] Nishimura Kohei, Fukagawa Tatsuo, Takisawa Haruhiko, Kakimoto Tatsuo, Kanemaki Masato (2009). An auxin-based degron system for the rapid depletion of proteins in nonplant cells. Nature Methods.

[R15] O'Brien Jacob, Hayder Heyam, Zayed Yara, Peng Chun (2018). Overview of MicroRNA Biogenesis, Mechanisms of Actions, and Circulation. Frontiers in Endocrinology.

[R16] Palumbos Sierra D., Popolow Jacob, Goldsmith Juliet, Holzbaur Erika L. F. (2025). Autophagic stress activates distinct compensatory secretory pathways in neurons. Proceedings of the National Academy of Sciences.

[R17] Petratou Dionysia, Fragkiadaki Persefoni, Lionaki Eirini, Tavernarakis Nektarios (2024). Assessing locomotory rate in response to food for the identification of neuronal and muscular defects in C. elegans. STAR Protocols.

[R18] Rivard Laura, Srinivasan Jagan, Stone Allison, Ochoa Stacy, Sternberg Paul W, Loer Curtis M (2010). A comparison of experience-dependent locomotory behaviors and biogenic amine neurons in nematode relatives of Caenorhabditis elegans. BMC Neuroscience.

[R19] Vidigal Joana A (2020). AGO unchained Canonical and non-canonical roles of Argonaute proteins in mammals. Frontiers in Bioscience.

[R20] Sawin Elizabeth R, Ranganathan Rajesh, Horvitz H.Robert (2000). C. elegans Locomotory Rate Is Modulated by the Environment through a Dopaminergic Pathway and by Experience through a Serotonergic Pathway. Neuron.

[R21] Shang Renfu, Lee Seungjae, Senavirathne Gayan, Lai Eric C. (2023). microRNAs in action: biogenesis, function and regulation. Nature Reviews Genetics.

[R22] Sharma Nidhi, Marques Filipe, Kratsios Paschalis (2024). Protocol for auxin-inducible protein degradation in C. elegans using different auxins and TIR1-expressing strains. STAR Protocols.

[R23] Stiernagle T (2006). Maintenance of C. elegans.. WormBook.

[R24] Taylor Seth R., Santpere Gabriel, Weinreb Alexis, Barrett Alec, Reilly Molly B., Xu Chuan, Varol Erdem, Oikonomou Panos, Glenwinkel Lori, McWhirter Rebecca, Poff Abigail, Basavaraju Manasa, Rafi Ibnul, Yemini Eviatar, Cook Steven J., Abrams Alexander, Vidal Berta, Cros Cyril, Tavazoie Saeed, Sestan Nenad, Hammarlund Marc, Hobert Oliver, Miller David M. (2021). Molecular topography of an entire nervous system. Cell.

[R25] TOPS B.B.J., PLASTERK R.H.A., KETTING R.F. (2006). The Caenorhabditis elegans Argonautes ALG-1 and ALG-2: Almost Identical yet Different. Cold Spring Harbor Symposia on Quantitative Biology.

[R26] Van Wynsberghe Priscilla M, Kai Zoya S, Massirer Katlin B, Burton Victoria H, Yeo Gene W, Pasquinelli Amy E (2011). LIN-28 co-transcriptionally binds primary let-7 to regulate miRNA maturation in Caenorhabditis elegans. Nature Structural & Molecular Biology.

[R27] Vidigal Joana A (2020). AGO unchained Canonical and non-canonical roles of Argonaute proteins in mammals. Frontiers in Bioscience.

[R28] Yesbolatova A, Saito Y, Kitamoto N, Makino-Itou H, Ajima R, Nakano R, Nakaoka H, Fukui K, Gamo K, Tominari Y, Takeuchi H, Saga Y, Hayashi KI, Kanemaki MT (2020). The auxin-inducible degron 2 technology provides sharp degradation control in yeast, mammalian cells, and mice.. Nat Commun.

